# Skeletal fluorosis: a rare cause of diffuse bone condensation

**DOI:** 10.11604/pamj.2017.26.186.11743

**Published:** 2017-03-30

**Authors:** Zeineb Alaya, Walid Osman

**Affiliations:** 1 Department of Rheumatology, Farhat Hached Hospital, Faculty of Medicine of Sousse, Sousse, Tunisia; 2 Department of Orthopaedics, Sahloul Hospital, Faculty of Medicine of Sousse, Sousse, Tunisia

**Keywords:** Bone condensation, skeletal fluorosis, radiography

## Image in medicine

A 69-year-old man, native of metlaoui in Tunisia, presented to the Department of Rheumatology at our institution with 10 years of non-inflammatory rachialgia with polyarthralgia. On examination, the patient had no fever or joint swelling, his teeth had brown strains and rough, there was diffuse tenderness at the cervical and lumbar spine and neurological examination was normal. Standard radiographs revealed diffuse bone condensation involving all vertebrae of the cervical, dorsal (A) and lumbar (B) spine, the skull (C), the pelvis (D) and the limbs. The origin of the patient, the brownish color of the teeth and the radiological appearance led to suspicion of fluoride poisoning, which was confirmed by the very high blood fluoride and urine dosage. Skeletal fluorosis is a rare toxic osteopathy characterized by massive bone fixation of fluoride. Its origin is dominated in the countries of North Africa by hydro-telluric poisoning. Its severity lies in the development of skeletal deformities and neurological complications. Management of fluorosis generally focuses on symptom treatment.

**Figure 1:**
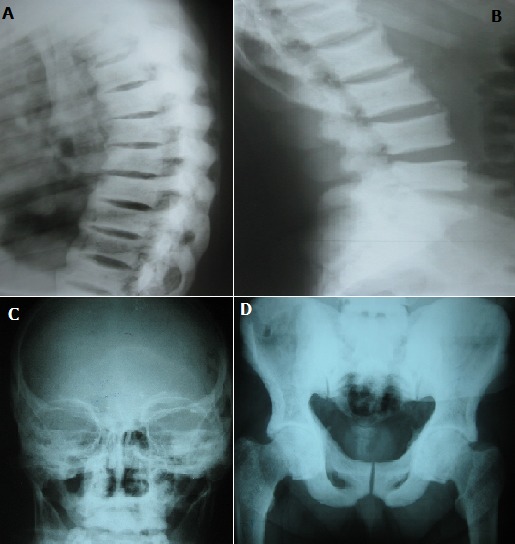
(A) X-ray of the dorsal spine in profile: diffuse bone condensation; (B) X-ray of lumbar spine in profile: diffuse bone condensation; (C)X-ray of the skull: diffuse bone condensation, (D) X-ray of the pelvis: osteosclerosis of the pelvis

